# A Comparative Study of Average, Linked Mastoid, and REST References for ERP Components Acquired during fMRI

**DOI:** 10.3389/fnins.2017.00247

**Published:** 2017-05-05

**Authors:** Ping Yang, Chenggui Fan, Min Wang, Ling Li

**Affiliations:** Key Laboratory for NeuroInformation of Ministry of Education, High-Field Magnetic Resonance Brain Imaging Key Laboratory of Sichuan Province, Center for Information in Medicine, School of Life Science and Technology, University of Electronic Science and Technology of ChinaChengdu, China

**Keywords:** ERP, REST reference, average reference, linked mastoid, N1, P300, statistical parametric scalp mapping (SPSM)

## Abstract

In simultaneous electroencephalogram (EEG) and functional magnetic resonance imaging (fMRI) studies, average reference (AR), and digitally linked mastoid (LM) are popular re-referencing techniques in event-related potential (ERP) analyses. However, they may introduce their own physiological signals and alter the EEG/ERP outcome. A reference electrode standardization technique (REST) that calculated a reference point at infinity was proposed to solve this problem. To confirm the advantage of REST in ERP analyses of synchronous EEG-fMRI studies, we compared the reference effect of AR, LM, and REST on task-related ERP results of a working memory task during an fMRI scan. As we hypothesized, we found that the adopted reference did not change the topography map of ERP components (N1 and P300 in the present study), but it did alter the task-related effect on ERP components. LM decreased or eliminated the visual working memory (VWM) load effect on P300, and the AR distorted the distribution of VWM location-related effect at left posterior electrodes as shown in the statistical parametric scalp mapping (SPSM) of N1. ERP cortical source estimates, which are independent of the EEG reference choice, were used as the golden standard to infer the relative utility of different references on the ERP task-related effect. By comparison, REST reference provided a more integrated and reasonable result. These results were further confirmed by the results of fMRI activations and a corresponding EEG-only study. Thus, we recommend the REST, especially with a realistic head model, as the optimal reference method for ERP data analysis in simultaneous EEG-fMRI studies.

## Introduction

In electroencephalogram (EEG) and event-related potential (ERP) research, the reference issue is an important problem. Previous studies investigated the effects of different references on simulated or real EEG/ERP data, showing that the voltage of the scalp potentials, power spectra, EEG coherence, connectivity configuration, DMN configuration, and even the polarity of some electrodes were changed by the adopted reference methods (Joyce and Rossion, 2005; Marzetti et al., 2007; Yao et al., 2007; Qin et al., 2010). Moreover, the statistical parametric scalp mapping (SPSM), which is the scalp distribution of the significant statistical difference between two conditions, varied depending on the adopted references (Tian and Yao, 2013). Therefore, the choice of different references might cause data misinterpretation within the same experiment.

Typically, the EEG/ERP references include average reference (AR), digitally linked mastoid (LM), vertex reference (CZ), and REST reference (Yao, 2001). AR is the most popular choice in ERP studies, since it uses the average of channels as reference and it is unbiased to any electrode position. However, an inadequate spatial sampling (e.g., sparse electrode array) can affect the AR data and the underlying source estimation (Lantz et al., 2003). LM, with the average of left and right mastoids as reference, is another popular reference method for ERP studies, since the LM is suggested to be far from all brain sources and thus could be treated as a zero potential point. Also, LM is independent of electrode montages, which facilitates the comparison of results from different laboratories with different electrode caps. However, using simulated data, previous studies showed that AR and LM references lead to significant distortion of scalp power distribution and scalp network structure (Yao et al., 2005; Qin et al., 2010). This occurs because using scalp recordings as reference, like AR and LM, would bring their own physiological dynamic signals into the EEG signal and thus affect the spatial and temporal aspects of the EEG signal (Yao, 2001; Thatcher, 2012). To minimize the effect of physical reference on EEG signals, Yao proposed a reference electrode standardization technique (REST) which calculated a reference point at infinity (Yao, 2001).

The REST is based on the fact that the EEG source estimates are reference-free (Geselowitz, 1998; Yao, 2001; Michel et al., 2004), so the scalp potential topography can be unambiguously reconstructed by a set of known generator sources for a given head model (forward solution). Therefore, Yao proposed a non-unique equivalent dipole source model, which assumed an equivalent source distribution (ESD) on the cortical surface. The ESD and a proposed three-concentric-sphere head model were used to compute a transfer matrix. Then, the transfer matrix can be used to rereference scalp potentials to an infinity reference (Yao, 2001). Please note that, the non-unique equivalent dipole source model is used to calculate the transfer matrix rather than to solve the EEG inverse problem. So that the transfer matrix is independent of the actual neuronal generators, and the REST reference is independent of the actual EEG data. Furthermore, this infinity reference (REST) is considered to be located far from all brain sources and scalp electrodes, and thus it induces a small effect on EEG signals. Previous studies have demonstrated that REST reference can approximately recover the EEG temporal waveform, power spectrum (Yao, 2001), EEG coherence (Marzetti et al., 2007), EEG connectivity patterns (Chella et al., 2016), and EEG network configuration (Qin et al., 2010). Also, simulation studies using a concentric three-sphere head model and a realistic head model (Zhai and Yao, 2004) have confirmed the accurateness of the REST reconstruction, even when a low-density montage was used (Liu et al., 2015). According to the REST calculation, its validity depends on the leadfield matrix. Thus, the limitations of REST are the electrode density and the accuracy of the head model (Yao, 2001; Nunez, 2010).

In the present study, we collected simultaneous electroencephalography and functional magnetic resonance imaging (EEG-fMRI) data with a classical working memory paradigm. To determine the reference that best identifies neural activity and therefore be the basis of improved estimates of ERP features in the present EEG-fMRI recordings, we compared the reference effects of AR, LM, REST with the sphere head model (RESTs), and REST with the realistic head model (RESTr) on the task-related ERP effects and its distribution (e.g., discrimination-related posterior N1, VWM load-related parietal P300, and so on), which is the most concern in cognitive neuroscience research and was confirmed to be affected by the reference choice (Kayser et al., 2007; Tian and Yao, 2013). We hypothesized the change of reference methods would alter the task-related ERP effect itself (measured by statistical significance) and especially its scalp distribution (measured by SPSM).

ERP cortical source estimations were used as the golden standard in this comparison, since the underlying neural sources are the same no matter what reference is actually adopted (Geselowitz, 1998; Yao, 2001; Michel et al., 2004). Please note that, the REST approach is independent of the actual EEG data, thus the REST does not give any special advantage over the other electrode references when EEG source estimation is performed. In more detail, we localized the ERP of interest source generators and got source activities for each source region (inverse solution), then the source activities can be projected back to scalp voltage (forward solution). According to scalp topographical maps at the respective latency of the peak source intensities, we can indicate the contribution of a given source to the scalp responses at specific electrode sites (Bledowski, 2004; Bledowski et al., 2006). Thus, the observed task-related differences in the peak source intensities can be considered responsible for task-related differences in the ERP at that electrode sites (Mitzdorf, 1985; Bledowski, 2004; Bledowski et al., 2006; Kayser et al., 2007). So, we computed the distribution of the task-related ERP effect on the scalp (SPSM) for each reference method, and compared them to the distribution of the task-related source activities acquired from ERP source regions and the corresponding fMRI activations. In addition, we assessed the reference effect on the EEG-only data analysis which was collected during the same task but without active fMRI recordings. This could provide supportive evidence for reference selection in EEG-fMRI studies.

Although, AR and LM are the most popular reference methods for the ERP analysis of simultaneous EEG-fMRI studies (Huettel et al., 2004; Bledowski et al., 2006; Novitskiy et al., 2011; Castelhano et al., 2014; Chun et al., 2016), they might alter the ERP results by bringing physical interference into the EEG signal as previously mentioned. The REST overcomes this problem by calculating a reference point at infinity but is limited with the electrode density and the accuracy of the head model. Since the individual structure image and realistic electrode positions were available, and the number of electrodes is sufficient (64-channels), we speculate that REST with the realistic head model (RESTr) is the best option in simultaneous EEG-fMRI studies.

## Materials and methods

### Subjects

Eighteen right-handed subjects (9 females), age 19–27 (mean age = 21.9 years, standard deviation = 2 years), participated in the EEG-fMRI study for monetary compensation. Another group of 14 right-handed subjects (3 females), age 21–28 (mean age = 23 years), were recruited for the EEG-only study. Subjects were recruited at the University of Electronic Science and Technology of China. All the subjects had no history of neurological problems and had normal color vision. An informed consent form was signed by each subject before the experiment. The study was approved by the local ethics committee for the Protection of Human Subjects for the University of Electronic Science and Technology of China. The methods were carried out in accordance with the approved guidelines and all experiments conformed to the declaration of Helsinki.

### Procedure

**Figure 1:**
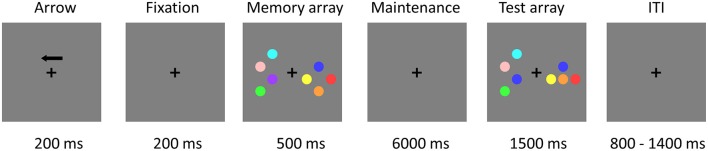
**Trial demonstration of the change detection task in the EEG-fMRI study**. Subjects were asked to maintain two or four objects in an attended visual side for 6 s and then to indicate whether the test array matched or mismatched the object colors in the memory array, regardless of object location. Independent EEG study without simultaneous fMRI recordings used the same paradigm, but the VWM maintenance interval was changed from 6,000 to 900 ms.

The stimuli consisted of nine disks with highly discriminable colors, including red, yellow, blue, green, cyan, purple, pink, orange, and carmine. The diameter of each disk was 2.2 cm and the distance between disks was at least 3.8° (center to center). Figure 1 illustrates an example of the change detection paradigm (Li et al., 2011). The trial began with a 200-ms black fixation cross and an arrow, which instructed subjects to attend and memorize the items in the corresponding visual field. A fixation cross was then presented alone for 200 ms. Following that, subjects were presented with a memory array for 500 ms, consisting of the same number of disks in the left and right visual fields, with 2 or 4 disks per hemi-field. Within each hemi-field, the disks were randomly selected without repetition from the nine potential disk colors. The disks were randomly located on an invisible 4 × 3 matrix (5.5° × 4.2°) in both visual fields. After the memory array presentation, a black fixation cross was presented for the duration of the 6,000 ms maintenance interval which allowed us to separate the activation related to the encoding, maintenance, and retrieval phases of the VWM task (Pessoa et al., 2002; Ranganath, 2006). Subsequently, the test array remained on the screen for up to 1,500 ms, during which subjects responded with a button press, indicating whether the colors of the disks in the attended hemi-field were the same or different from those in the memory array regardless of object locations. Only one colored disk changed its location from the memory to the test array for the *location change condition* and none of the objects changed their original location for the *location repeat condition*. After the test array, the inter-trial interval (ITI) varied between 800 and 1,400 ms (average ITI was 1.1 s).

In the present experiment, subjects were instructed to remember the color and disregard the location of the disks in the attended hemi-field. Subjects were required to maintain central fixation throughout the recordings and to respond as quickly as possible. Subjects used their right hands to press button 1 when the color of all the disks in the memory array was the same as that of the test array in the attended visual field, and button 2 when the color of a disk was changed.

The EEG-only study used the same paradigm, but the VWM maintenance interval was changed from 6,000 to 900 ms (Li et al., 2011).

### EEG and fMRI recording

Subjects performed the task inside the MRI scanner (GE Signa 3.0 T) with simultaneous EEG and MRI recordings. The sampling clocks of the EEG and MRI systems were synchronized by means of the Syncbox (BrainProducts).

EEG signals were collected using a 64-channel fMRI-compatible Neuroscan Maglink System with Ag/AgCl electrodes placed according to the international 10/20 electrode placement standard. Vertical and horizontal electrooculogram (EOG) were recorded with electrodes above and on the outer canthi of the left eye. The electrocardiograms (ECGs) were recorded with a pair of electrodes above and below the left sternum. EEG data were sampled at 1,000 Hz and the electrode impedances were kept under 10 KΩ throughout the experiment. The amplifier gain was 150 and the analogic bandpass filter was set at 0–200 Hz. The AFz electrode site served as the ground electrode and an electrode between Cz and Pz served as reference.

Functional MR images were acquired with a gradient echo planar imaging (EPI) sequence with the following scanning parameters: TR = 2,000 ms; TE = 30 ms; FA = 90°; FOV = 240 mm; matrix size = 64 × 64; voxel size = 3.75 × 3.75 × 4.4 mm^3^; 35 slices. The structural images were acquired with a high-resolution T1-weighted scan (voxel size = 1 × 1 × 1 mm^3^).

### EEG data preprocess and re-reference

EEG data preprocessing was performed using the CURRY 7 Neuroimaging Suite. The preprocessing step included filtering between 0.1 Hz (slope, which is the frequency range from complete attenuation to complete transfer, is 0.2 Hz) and 48 Hz (slope is 9.6 Hz), removing gradient artifacts (using average subtraction during each TR interval), removing ballistocardiogram artifacts (PCA based correction) and removing EOG artifacts (amplitude exceeding a ±60 μv threshold). Artifact-free EEG data was exported to MATLAB and EEGlab for further analysis.

The continuous EEG data were segmented into test array locked epochs (from −200 to 1,000 ms relative to the test array onset). Trials in which the EEG activities exceeded 100 μV and contained incorrect responses were rejected. The remaining trials were re-referenced to AR, LM, RESTs, and RESTr references. The AR reference used the average of all channels as reference, whereas the LM used the average of left and right mastoid as reference.

For the RESTs reference, a three-concentric-sphere head model was reconstructed with the radii of the three concentric spheres: 0.87 (inner radius of the skull), 0.92 (outer radius of the skull), and 1.0 (radius of the head). The conductivities were 1.0 (brain and scalp) and 0.0125 (skull). The coordinates of the electrodes are automatically normalized to the spherical surface with radius 1.0. The lead field matrix was calculated from 3,000 radical cortical dipoles distributing on the spherical surface using the LeadField.exe (in REST software). Finally, EEG data, electrode positions and lead field matrix were imported into REST software (http://www.neuro.uestc.edu.cn) and then the REST reference was applied to the EEG data.

For the RESTr reference, (1) we extracted the cortex surface from subject's MRI images using the BrainVISA (version 4.3 http://brainvisa.info/); (2) we then reconstructed a 3-shell realistic BEM head model on the cortex surface, by means of Brainstorm (http://neuroimage.usc.edu/brainstorm); (3) projected the electrode positions on the scalp surface, and then modified the electrode positions based on the head shape and EEG gel artifact observed in the structural image; (4) the cortex surface was down-sampled to 3,000 vertices; (5) the transfer lead field matrix was calculated by the above-mentioned electrode positions and head model using the OpenMEEG boundary element method (Gramfort et al., 2010), where conductivities for the scalp, skull, and brain were 1.0, 1/80, and 1.0 separately. Sample dipoles were positioned at each vertex, with their directions constrained to be perpendicular to the cortical surface; (6) EEG data, electrode positions and lead field matrix are imported into REST software and then the REST reference was applied to the EEG data. Above steps were perform for each subject.

### ERP analysis

By inspecting the grand average ERP waveforms and the topographic maps, two ERP components of interest were examined. N1 was defined as a negative deflection in the 150–200 ms time window and P300 was defined as a positive deflection in the 300–600 ms time window after the test array onset. The N1 peak amplitude and latency were measured and averaged across the left (P5, P3, P1) and right (P2, P4, P6) parietal electrodes sites as two separate clusters. P300 mean amplitudes were measured between 300 and 600 ms and averaged across the central-parietal electrode cluster (CP1, CP2, P1, PZ, P2).

To evaluate the reference effects on ERP components, we applied a four-way repeated measure analysis of variance (ANOVA) with reference method, memory load, cue side, and location change/repeat as factors for N1 and P300 components, separately. After that, for each reference data, we applied a three-way repeated ANOVA with memory load, cue side, and location change/repeat as factors for N1 and P300 components, separately. Such analysis investigates whether the experimental effect (significant differences across experimental conditions) would be changed by the adopted references.

### SPSM analysis

We computed a *t-*test of the N1 amplitude (peak value between 150 and 200 ms) between *location repeat* and *location change* condition for each electrode. The resulting *P*-value was described on a topography map with a threshold of 0.05 to form the statistical parametric scalp mapping (SPSM). The above processes were performed for each reference data. Such analysis seeks to investigate whether the scalp distributions of experimental effect (significant differences across experimental conditions) would be changed by the adopted references.

### Source analysis

Source reconstruction of individual ERP data was performed using the Brainstorm 3.0 software. We used the standard MNI template ICBM152, which is consistent with the MNI template used in the fMRI analysis, to create the head model by using the boundary element method. The cortical current maps were computed from the ERP time series using the weighted minimum norm estimate (wMNE) inverse solution for each condition in each subject separately, as well as for the grand average condition (combined for eight conditions) in each subject. The source orientation was constrained to be normal to the cortical surface. Subject-wise cortical current maps were normalized (*z*-score) with the baseline period (−200 to −1 ms). The group-wise cortical maps were computed by the average of *z*-score across all subjects in each condition and the grand averaged condition, and then spatially smoothed with a 6 mm FWHM Gaussian filter.

For each potential source, we extracted the source activities for *location repeat* and *location change* conditions. Furthermore, the source activities were projected back to scalp voltage, and the topographical maps were calculated at the respective peak latency in order to assess the contribution of the current source to the scalp voltage. To assess whether the sources responsible for the location-related effect as measured on the posterior N1 component, the mean source intensities (±10 ms around the peak) were calculated for each condition in each subject and then compared using a three-way ANOVA. The location-related fMRI activations were also treated as the potential sources of N1 and were processed using the above-stated steps.

Please note that we did not calculate the P300 source nor extract the P300 source waveforms. This was because the generators of P300 are widely distributed in space and time (Kok, 2001), thus it is hard to use the P300 sources to verify the distribution pattern of VWM load-related effect on P300. Our fMRI results also showed the VWM load-related activations were widely distributed at frontal, parietal, temporal, and occipital cortices.

### fMRI preprocessing and analysis

fMRI data preprocessing was performed using statistical parametric mapping software (SPM12, http://www.fil.ion.ucl.ac.uk/spm) for each subject. The first five EPI volumes of the fMRI images were discarded for signal stabilization. fMRI data preprocessing included slice timing correction, three-dimensional motion correction, co-registration to individual anatomical images, normalization to the Montreal Neurological Institute (MNI) reference space (3 × 3 × 3 mm^3^), and spatial smoothing with an 8 mm Gaussian kernel (full-width at half-maximum). One session from one subject with a total vector motion >2 mm or rotation >2° was excluded from further analysis.

For the first level statistical analyses, a general linear model (GLM) was constructed for each subject's observed fMRI time course. Three time points (representing the onsets of arrow, delay, and test arrays) were defined for each condition and convolved with a canonical hemodynamic response function (HRF) to form regressors of the design matrix (Gazzaley et al., 2007; Robitaille et al., 2010; Passaro et al., 2013). Moreover, six additional spatial movement regressors were added to the design matrix. The memory array period was not modeled in the design matrix since the time interval between the arrow and the memory array was too small. Thus, the regressor of the arrow was used to represent both the arrow and memory periods in the present study. The data and models for each individual subject were high-pass filtered to a cutoff of 1/128 Hz and pre-whitened with a fitted autoregressive model [AR (1)].

For the second level statistical analyses, VWM retrieval-related maps were compared using a one-sample *t-*test, contrasting the combined activation across conditions during the retrieval phase with the fixation baseline. The retrieval-related map was thresholded at *P* < 0.05 (FDR corrected) and cluster size >45 voxels, and then used as a prior mask for the following statistics. Individual subject contrast images for each condition, during the VWM retrieval phase, were entered into a random-effect model with a 2 (load 2 vs. 4) × 2 (left vs. right visual field) × 2 (location repeat vs. change) ANOVA using GLM_Flex2 (http://mrtools.mgh.harvard.edu/index.php/GLM_Flex). Masked by the retrieval-related map, we reported clusters >15 contiguous voxels, at a voxel-wise threshold of *P* < 0.005 (uncorrected, cluster size corrected to *P* < 0.01 using the AlphaSim; Forman et al., 1995), for location effects.

Regions of interest (ROIs) were defined based on the multi-subject statistical maps. A 6-mm radius sphere (centered around the peak activation of each cluster) was drawn as a ROI, by means of MarsBar software (http://marsbar.sourceforge.net). The values of each ROI were analyzed using a three-way repeated-measures ANOVA as described above.

We used the fMRI location-related clusters as regional sources to obtain the source waveform and scalp projection. The peak intensities were calculated for each condition in each subject and then compared using a three-way ANOVA, for each ROI (same process in Source Analysis).

### EEG-only recordings (collection, preprocessing, and analysis)

EEG-only was recorded using a 128-channel EGI HydroCel GSN (EGI, Eugene, OR, USA) electrode cap with electrodes placed according to the international 10/20 electrode placement standard. EEG signals were recorded using NetStation 4.1.2 with a Net Amps 300 amplifier (Electrical Geodesic Inc., EGI, Eugene, Oregon, USA). The online reference electrode was the Cz (129th) and the ground reference had a centroparietal location. All electrode impedances were kept well below 50 kΩ. EEG was digitized at 1,000 Hz with an amplifier band-pass of 0.1–48 Hz.

EEG data were processed off-line using Net Station Waveform Tools and Matlab. The continuous EEG data were filtered by a two-way FIR bandpass filter from 0.1 to 48 Hz (eegfilt.m from EEGLAB toolbox), and were segmented into test array locked epochs (from −200 to 1,000 ms relative to the test array onset). For each segments, channels with amplitudes exceeding 200 μV were marked as undesirable and replaced through the interpolation of neighboring electrodes. EOG and significant muscle artifacts were excluded by automatic artifact rejection (±100 μV). EEG epochs containing incorrect button presses and eye movements were excluded. The data was baseline corrected using the 200 ms before the onset of the memory array. EEG epochs were then averaged across trials according to load (two and four), visual side (left and right), and location repeat/change conditions. For each subject, at least 32 trials were included for each condition. The remaining trials were re-referenced to AR, LM, and RESTs reference. Since LM reference distorted the grand-average ERP shape, it will not be used for subsequent analyses.

ERP analysis refers to the analysis of ERP data in the EEG-fMRI study. N1 and P300 were selected as the ERP components of interest. The N1 peak amplitude and latency were measured and averaged across the left (P7, P3, PO3, and 66) and right (P8, P4, PO4, and 84) parietal electrode sites as two separate clusters. P300 mean amplitudes were measured between 300 and 600 ms and averaged across the central-parietal electrode cluster (CP1, CP2, C1, CZ, C2, 7, 106, 31, 80). SPSM analysis for the N1 component measured by the *t-*test between *location repeat* and *location change* conditions using the peak N1 amplitude (150–200 ms interval), for each electrode separately. Source analysis refers to the Source Analysis in the EEG-fMRI study.

### Statistical analysis

Statistical analyses were performed using SPSS Statistics Release 19 (IBM, Somers, NY, USA) General Linear Model. Bonferroni corrections were performed for multiple comparisons, and Greenhouse-Geisser corrections were performed for non-sphericity data where necessary. *Post-hoc* multiple-comparison tests were performed where appropriate.

## Results

### ERP effects

**Figure 2:**
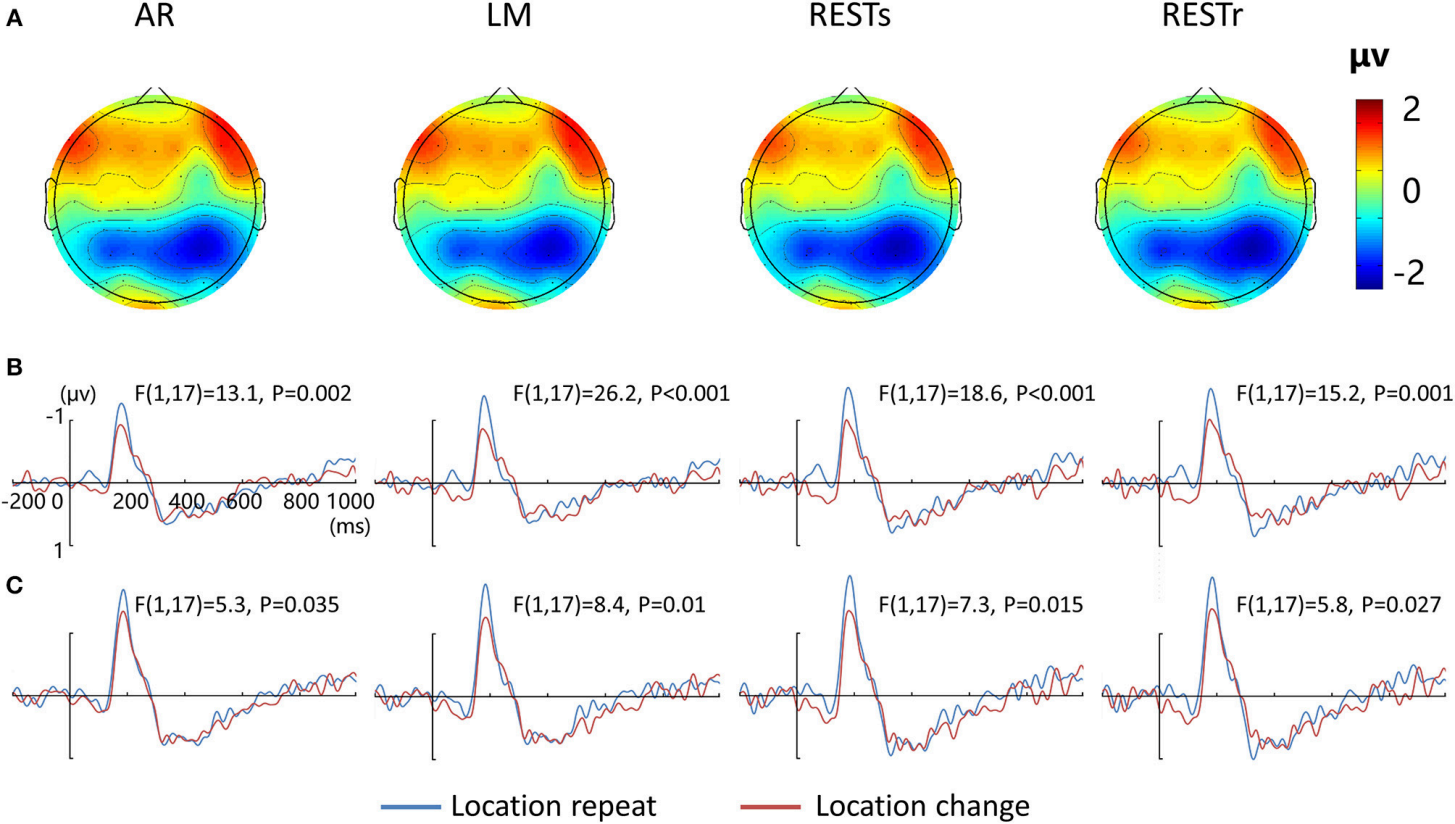
**EEG-fMRI study. (A)** Voltage topographies of N1 peaks for four references. ERP waveform for location repeat and location change conditions at **(B)** left (P5, P3, P1), and **(C)** right (P2, P4, P6) parietal electrode sites, for four references separately.

Figure 2A depicts the topographic map of the test array locked to the N1 component at 187 ms, which was defined by the peak of the grand-average ERP data, for AR, LM, RESTs, and RESTr references separately. Consistent with previous studies, the choice of different references would not change the spatial distribution pattern of the voltages (Yao et al., 2007). Figures 2B,C depict the test array locked ERPs for the *location repeat* and *location change* conditions at left and right electrode clusters, calculated for four references separately.

For the N1 amplitude at left electrode clusters, a four-way repeated measures ANOVA revealed a location effect [*F*_(1, 17)_ = 20.5, *P* < 0.001], as well as an interaction between reference method and location effect [*F*_(3, 15)_ = 3.9, *P* < 0.05]. For the N1 amplitude at right electrode clusters, we detected a location effect [*F*_(1, 17)_ = 7.0, *P* < 0.05] and an interaction between reference method and location effect [*F*_(3, 15)_ = 3.9, *P* < 0.05].

After that, we performed three-way repeated measures ANOVAs for N1 amplitude at the left and right electrode clusters, calculated for the four references separately. N1 amplitude was significantly larger during the *location repeat* compared with the *location change* condition at both left and right electrode clusters, with corresponding *F*- and *P*-values for different references described in Figure 2.

For the N1 latency, neither reference effects nor interaction effects between reference and other factors were detected, both at left or right electrode clusters (all Ps > 0.1). When we examined each type of reference data, we detected delayed N1 latency for left-view stimuli compared to right-view stimuli at left electrode clusters, for AR [*F*_(1, 17)_ = 10.6, *P* = 0.005], RESTs [*F*_(1, 17)_ = 6.3, *P* = 0.022], and RESTr [*F*_(1, 17)_ = 5.8, *P* = 0.028]. Furthermore, we detected delayed N1 latency for load 2 compared with load 4 at right electrode clusters, for AR [*F*_(1, 17)_ = 11.5, *P* = 0.003], MM [*F*_(1, 17)_ = 34.9, *P* < 0.001], RESTs [*F*_(1, 17)_ = 13.8, *P* = 0.002], and RESTr [*F*_(1, 17)_ = 9.3, *P* = 0.007].

**Figure 3:**
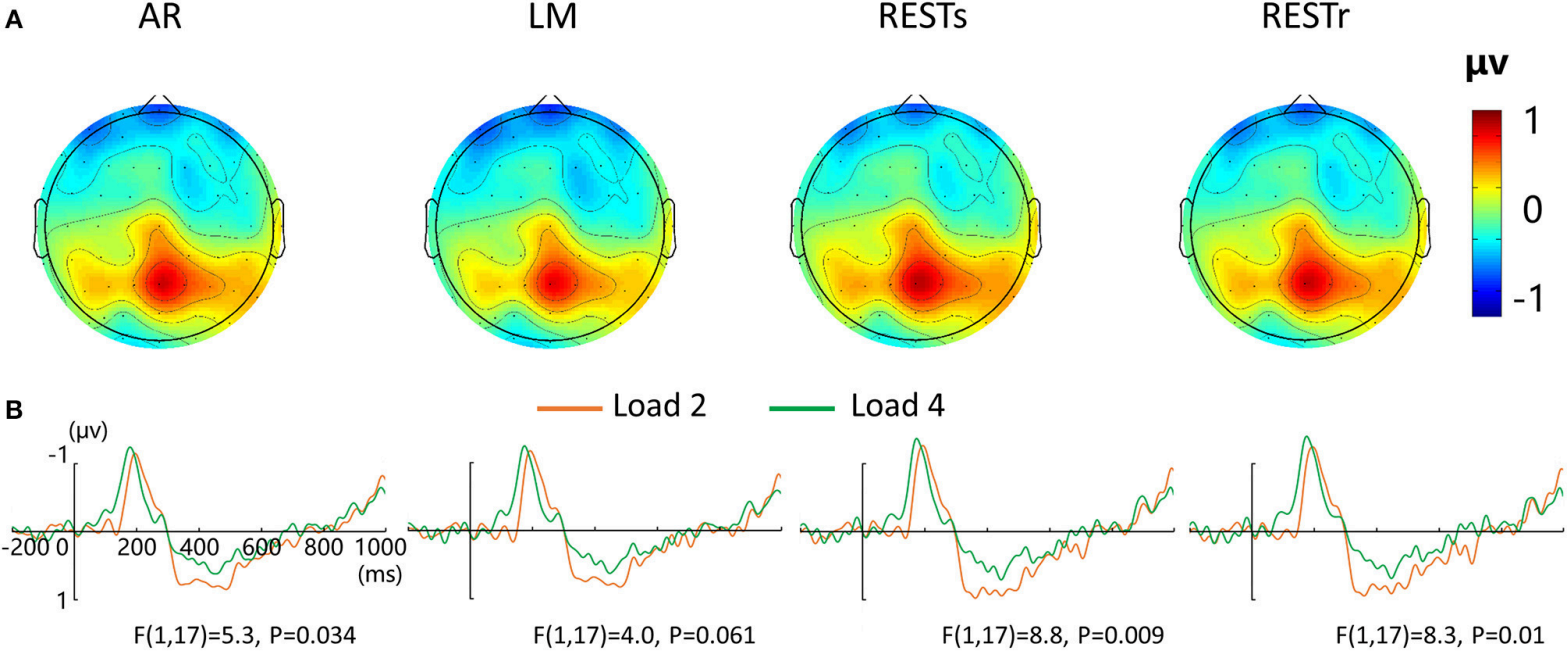
**EEG-fMRI study. (A)** Voltage topographies of P300. **(B)** ERP waveform for load 2 and load 4 conditions at central-parietal electrode cluster (CP1, CP2, P1, PZ, P2), for four references separately.

For the P300 amplitude, a four-way repeated measures ANOVA revealed a load effect [*F*_(1, 17)_ = 2.4, *P* < 0.05] and a marginally significant main effect of reference [*F*_(3, 15)_ = 2.8, *P* = 0.077]. After that, we performed a three-way repeated measures ANOVA for each type of reference data. P300 amplitude revealed a significant main effect of VWM load for AR and two REST data but a marginally significant load effect for LM data. The corresponding *F*- and *P*-values are described in Figure 3.

### SPSM results

**Figure 4:**
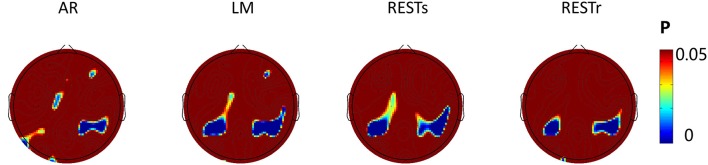
**EEG-fMRI study**. SPSM (location repeat vs. location change) of N1 peak calculated at 150–200 ms.

Since the N1 amplitude (peak value between 150 and 200 ms) revealed a significant difference between *location repeat* and *location change* conditions, we computed the significance level (*P-*value) for each electrode and presented the *P*-value on the topography map with a threshold of 0.05 (see Figure 4). For AR, the significant location effect was distributed at left posterior (P3, P5), right posterior (P2, P4, P6, CP2, CP4, CP6), frontal (FZ, F4), and left center (C1) electrodes sites. For LM, the significant location effect was distributed at left posterior (P3, P5, CP3, CP1), right posterior (P2, P4, P6, CP2, CP4, CP6, C6), left center (C1), and right frontal (F4) electrodes sites. For RESTs, the significant location effect was distributed at left posterior (P3, P5, CP3, CP1), right posterior (P2, P4, P6, CP2, CP4, CP6, C6), and left center (C1) electrodes sites. For RESTr, the significant location effect was distributed at left posterior (P3, P5, CP3, CP1) and right posterior (P2, P4, P6, CP2, CP4, CP6, C6) electrodes sites.

### Source results

**Figure 5:**
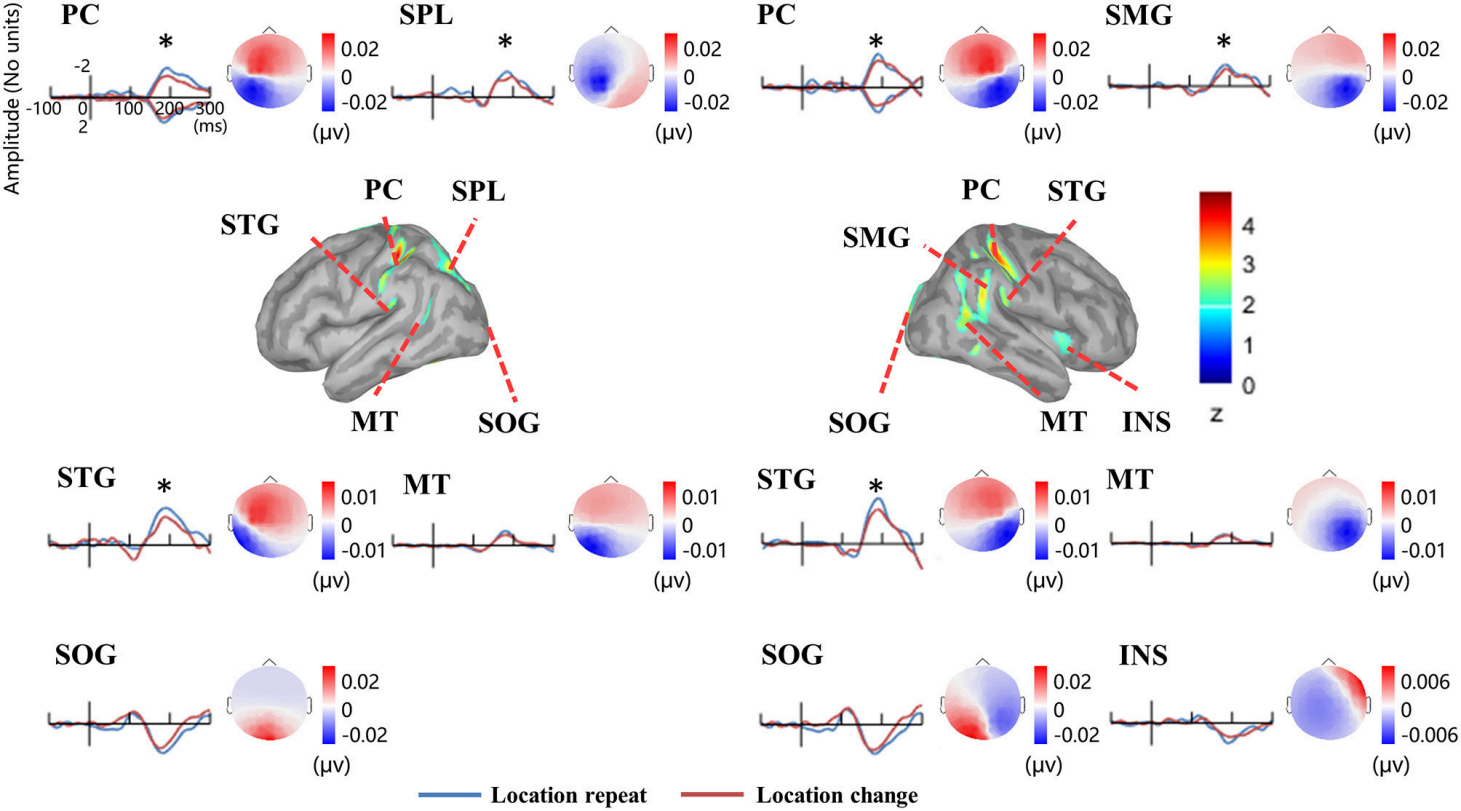
**EEG-fMRI study**. Time courses of the regional source activity for *location repeat* and *location change*, and the scalp projection at the peak intensity. Cortical sources of the posterior N1 component on the ICBM152 MNI template (middle). ^*^P < 0.05.

Figure 5 illustrates the group-wise cortical maps in the N1 time range. Active sources were defined as those containing at least 15 adjacent vertices exceeding a z-score of 2. The results revealed bilateral activations in postcentral gyrus (PC), superior temporal gyrus (STG), middle temporal gyrus (MT), and superior occipital gyrus (SOG), as well as left hemisphere activations in superior parietal lobule (SPL), and right hemisphere activations in supramarginal gyrus (SMG), and insula (INS; Table 1).

**Table 1:** **Sources of the N1 ERP component in the EEG-fMRI study**.

**Sources**	**H**	***x***	***Y***	***z***
Postcentral gyrus	L	−44	−34	55
	R	34	−36	59
Middle temporal gyrus	L	−47	−54	8
	R	47	−51	16
Superior temporal gyrus	L	−52	−33	17
	R	52	−29	20
SupraMarginal gyrus	R	57	−48	27
Insula	R	39	18	3
Superior parietal lobule	L	−22	−66	45
Superior occipital gyrus	L	−6	−103	12
	R	7	−92	18

*Regions showing the center of sources of the N1 component. Active sources with z-score >2 and adjacent vertices >15 are listed. (L, left; R, right; MNI coordinates are presented)*.

Analysis of the scalp projection indicated these sources contribute to a negative scalp ERP at bilateral parieto-occipital electrodes sites and a positive scalp ERP at bilateral fronto-central electrodes sites. Three-way ANOVAs showed significant location effects in bilateral PC and STG, as well as left SPL and right SMG (see Figure 5).

### fMRI results

**Figure 6:**
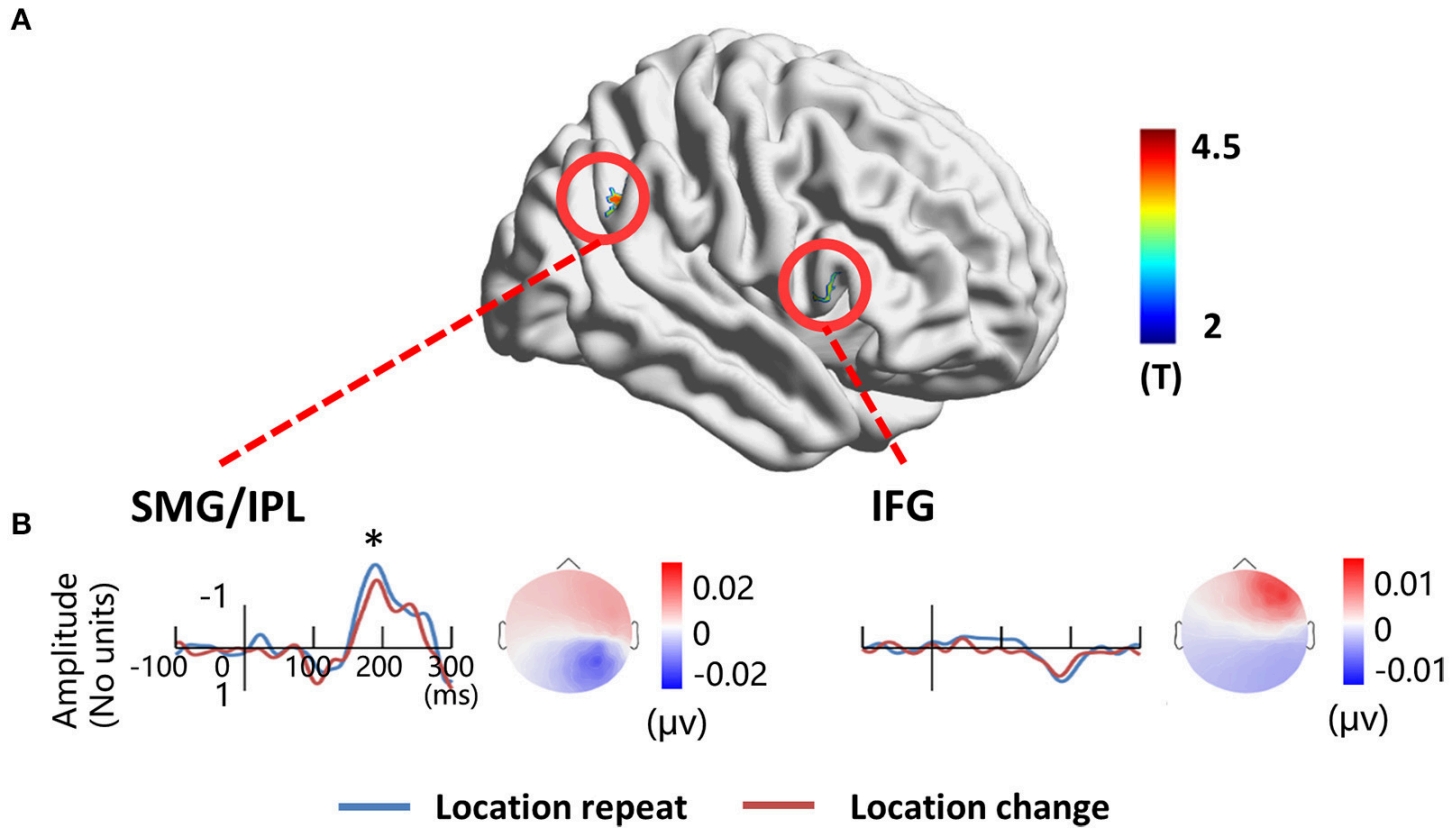
**EEG-fMRI study. (A)** location-related fMRI activation regions. **(B)** Source time courses of the right SMG/IPL and the right IFG for the *location repeat* and *location change* conditions, and the corresponding scalp projection at the peak latency. ^*^P < 0.05.

To localize the location-related brain regions, we contrasted activations of the *location repeat* with the *location change* conditions. The results showed significantly greater activations during the *location repeat* condition in the right SMG (BA 40) and right IFG (BA 45) compared to the *location change* condition. The results were projected onto a 3D surface using BrainNet (Xia et al., 2013) (Figure 6A).

The source in the right SMG revealed a negative deflection at 187 ms and the corresponding scalp projection indicated the right SMG source activity contributed mainly to the right posterior N1 component (Figure 6B). A three-way ANOVA showed a significant location effect [*F*_(1, 17)_ = 5.14, *P* < 0.05] on the source intensities. The source in the right IFG revealed a positive deflection at 187 ms, and this source activity contributed mainly to the right frontal positivity of ERP (Figure 6B). No significant main effects for location were detected (*P* > 0.1).

### Results of EEG-only study

**Figure 7:**
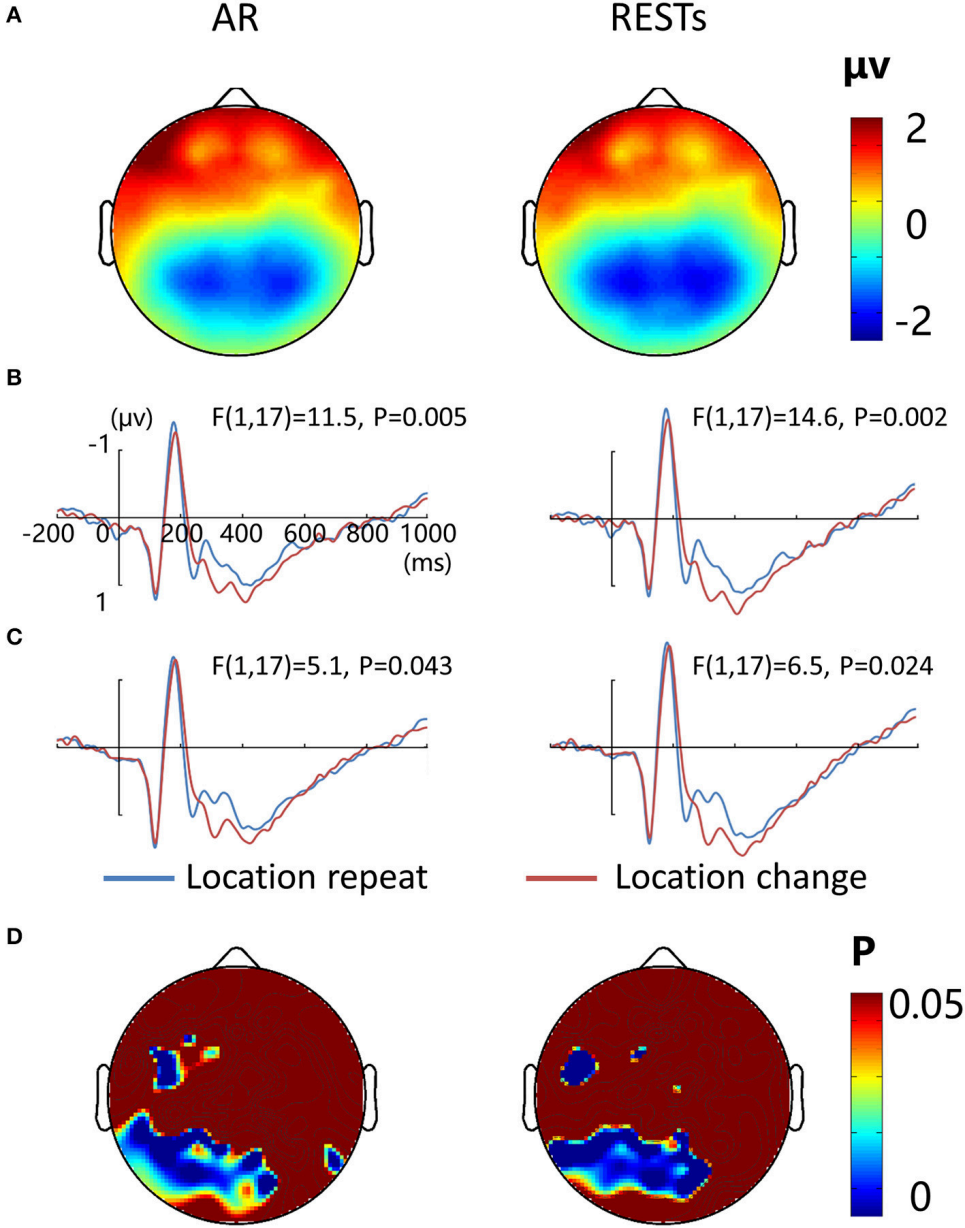
**EEG-only study. (A)** Voltage topographies of N1 peaks for AR and RESTs references. ERP waveform for *location repeat* and *location change* conditions at **(B)** left (P7, P3, PO3, and 66), and **(C)** right (P8, P4, PO4, and 84) parietal electrodes sites, for two references separately. **(D)** SPSM (location repeat vs. location change) of N1 peak calculated at 150–200 ms.

Figure 7A depicts the topographic map of the test array locked to the N1 component at 187 ms, which was defined by the peak of the grand-average ERP data, for AR and RESTs separately. Figures 7B,C depict the test array locked ERPs for the *location repeat* and *location change* conditions at left and right electrode clusters, calculated for each reference separately.

N1 amplitude was significantly larger during the *location repeat* compared with the *location change* condition at both left and right electrode clusters, with corresponding *F*- and *P*-values for different references described in Figure 7. For the N1 latency at the left posterior electrode cluster, we detected delayed N1 latency for low load compared to high load conditions [AR data: *F*_(1, 17)_ = 15.3, *P* = 0.002; RESTs data: *F*_(1, 17)_ = 17.4, *P* = 0.001], and for *location change* than *location repeat* conditions [AR data: *F*_(1, 17)_ = 20, *P* = 0.001; RESTs data: *F*_(1, 17)_ = 21.2, *P* < 0.001]. For the N1 latency at the right posterior electrode cluster, we detected delayed N1 latency for low load compared to high load conditions [AR data: *F*_(1, 17)_ = 32.6, *P* < 0.001; RESTs data: *F*_(1, 17)_ = 43, *P* < 0.001], and for *location change* than *location repeat* conditions [AR data: *F*_(1, 17)_ = 8, *P* = 0.014; RESTs data: *F*_(1, 17)_ = 5.2, *P* = 0.041].

The central P300 component showed higher amplitude for load 2 compared to load 4 conditions [AR data: *F*_(1, 17)_ = 68.5, *P* < 0.001; RESTs data: *F*_(1, 17)_ = 76.5, *P* < 0.001]. Furthermore, the *location change* elicted higher P300 amplitude load compared to *location repeat* conditions [AR data: *F*_(1, 17)_ = 5.1, *P* = 0.043; RESTs data: *F*_(1, 17)_ = 8.1, *P* = 0.14].

SPSM of N1 showed significant location effect at left frontal (19, F3, 39, 44) and bilateral posterior electrode sites (50–52, 56–60, 63–72, 74–77, 82–84, 89–90 electrodes), both for AR and RESTs data.

**Figure 8:**
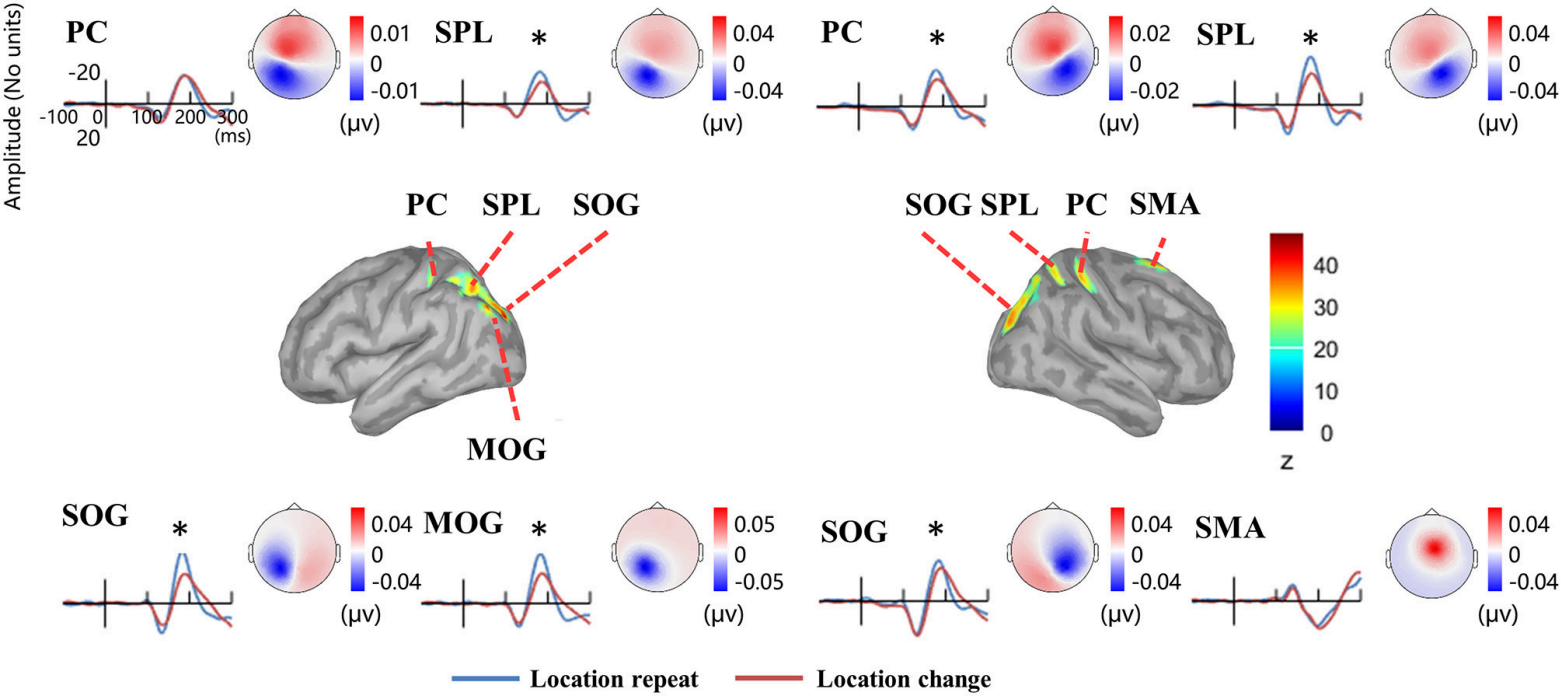
**EEG-only study**. Time courses of the regional source activity for location repeat and location change, and the scalp projection at the peak intensity. Cortical sources of the posterior N1 component on the ICBM152 MNI template (middle). ^*^P < 0.05.

Source analysis showed the N1 component generated in bilateral PC, SPL, and SOG, left MOG, as well as right SMA (Figure 8, Table 2). Analysis of the scalp projection indicated these sources, except right SMA, contribute to a negative scalp ERP at bilateral parieto-occipital electrode sites. Three-way ANOVAs showed location repeated enhancement in the peak source activities in bilateral SPL, SOG, left MOG, and right PC, as showed in Figure 8.

**Table 2:** **Sources of the N1 ERP component in the EEG-only study**.

**Sources**	**H**	***x***	***y***	***z***
Postcentral gyrus	L	−38	−37	56
	R	35	−39	62
Superior occipital gyrus	L	−24	−84	31
	R	24	−80	30
Superior parietal lobule	L	−32	−67	49
	R	33	−58	64
Middle occipital gyrus	L	−43	−75	36
Supplementary motor area	R	15	14	66

*Regions showing the center of sources of the N1 component. Active sources with z-score >20 and adjacent vertices >15 are listed. (L, left; R, right; MNI coordinates are presented)*.

## Discussion

The current study sought to identify the optimal referencing electrode procedure to study scalp ERPs recorded during fMRI scanning. We applied AR, LM, RESTs, and RESTr references to the ERP analysis within a simultaneous EEG-fMRI study and compared the reference effects on task-related ERP results which is a concern in cognitive neuroscience research and was confirmed to be affected by the reference choice (Kayser et al., 2007; Tian and Yao, 2013). ERP cortical source estimates were used as the golden standard in this comparison. It is because that the source localization of ERP components is reference-free, and also the implementation of the REST reference is independent of the actual EEG data. Results showed that the two REST references provided more integrated and reasonable results than that of AR and LM reference methods. These results were further confirmed by task-related fMRI activations and a corresponding EEG-only study.

### Reference effect on the ERP amplitude and latency

Similar topographic maps of N1 and P300 components were observed across four reference methods, which is consistent with previous studies reporting that the choice of different references would not change the spatial distribution pattern of the scalp voltages (Yao et al., 2007; Tian and Yao, 2013). However, in the present study, we did not find a main effect of reference methods on the N1 amplitude and latency, only a marginally significant main effect of reference methods on the P300 amplitude was observed. This is partly inconsistent with previous evidence showing that the ERP amplitude would be significantly altered by the adopted references (Kayser et al., 2007; Yao et al., 2007; Tian and Yao, 2013; Liu et al., 2015).

Actually, the amplitudes of N1 or P300 were small (about −1.3 uv for N1 and about 1 uv for P300) and showed high similarity across different references (Figures 2, 3). This may be due to the static magnetic field interference on the EEG signal collected in the MRI scanner (Toyomaki and Yamamoto, 2007). Additionally, MRI gradient artifacts and ballistocardiogram artifacts result in small voltages of EEG signal after a series of preprocessing compared to that collected outside the scanner (Srivastava et al., 2005). Since the original signal is very weak, it is difficult to detect the difference across different references.

### Reference effect on the task-related ERP

Although, the reference choice does not change the spatial distribution of ERP component, it might change the significant difference between two experimental conditions. For example, a prominent occipital vision vs. audition attentional effect was observed in REST and AR reference, but not in LM reference (Tian and Yao, 2013). Consistent with above findings, we observed an interaction between reference choice and location effect both at left and right electrode clusters for N1 amplitude. As shown in Figures 2B,C, although location repeated enhancement was observed at both left and right electrode clusters at each reference method, the significance level (*p*-value) had a slight difference with different reference methods.

For the load-related P300 amplitude, although no interaction between reference choice and VWM load was observed, the significance level of VWM load effect was altered by the adopted references (Figure 3B). Compared with AR data, two REST references increased the VWM load effect while the LM decreased or eliminated the VWM load effect [*F*_(1, 17)_ = 4.0, *P* = 0.061].

More importantly, we used multiple adjacent electrodes as ROI and used the mean value of ROI to test the task-related effect, which improves the signal-to-noise ratio (Keil et al., 2014) of data. This may be the possible reason why these different references showed slight effect on the outcome.

### Reference effect on the distribution of the task-related ERP

To assess the distribution of the experimental effects over the electrode sites, we performed the *t-*test of N1 amplitude between object location repeat and object location change conditions for each electrode and used the *p*-value to form the SPSM (statistical parametric scalp mapping) of N1 (Tian and Yao, 2013). The SPSM of N1 showed a distribution that is mostly the same but with a few differences, when using different references (Figure 4). In particular, we observed a significant location effect at the right posterior sites for four types of reference data, while less significance at left posterior sites for AR data. C1 and CP1 showed significant location effect for AR, LM, and RESTs data, but not for RESTr data. F4 showed a significant location effect for AR and LM data, but not for REST data.

To verify the distribution of the location effect, we localized the N1 sources and detected the generators related to the location effect as measured on the scalp. As several researchers stated that the EEG source estimates are independent of the EEG reference (Pascual-Marqui and Lehamann, 1993; Geselowitz, 1998; Yao, 2001), so it is reasonable to use the source distribution and source activities as the golden standard to infer the relative utility of different references. According to the scalp projection, source waveform, peak intensities latency, and the location effect on the peak intensities, we suggest that the generators in bilateral PC, SPL, STG, and right SMG were mainly responsible for the location effect for the bilateral posterior N1 component (Figure 5). These results suggest that LM and the two REST references provide the closest distribution pattern of the N1 SPSM to the source analysis. The relatively credible results of N1 SPSM from the LM referenced data may be due to the phenomenon that the potentials of two mastoids are actually near zero at the N1 topographic map (Figure 2A). In the source analysis, we did not detect the location effect at C1 and CP1 electrode for AR, LM, and RESTs data, and the location effect at the F4 electrode for AR and LM data.

By inspecting the SPSM of N1 in RESTs data and RESTr data, the RESTr seem provided a closer distribution to the location-related source distribution since the RESTs still revealed some significant points at C1 and CP1. Such result may support the improvement of accuracy of the REST reconstruction when a realistic head model (volume conductor) was used (Zhai and Yao, 2004; Liu et al., 2015).

It is worth noting that the head volume conduction effects can confound the topography and amplitude of ERPs at the scalp electrode. Thus, to accurately evaluate the cortical activity under the scalp EEG electrodes, a researcher should be encouraged to perform a source analysis which is able to take into account at least in part the head volume conduction effects. In the previous simulation studies, researchers have demonstrated the validity of REST even when the volume conductor differs from the true head model and even when the conductivity ratio was varied, showing that the relative error between the simulated EEG recordings and the EEG recordings referenced at infinity is greater reduced by REST compared with other commonly used references (Yao, 2001; Qin et al., 2010; Liu et al., 2015). However, even when the realistic MRI head model was used, it cannot mitigate the overlapping effects of the neural ionic currents at the scalp electrodes.

### fMRI supports

Since EEG and fMRI are two modalities related to the same neuronal activity (Logothetis et al., 2001), we used the task-related fMRI brain regions as sources to test if these sources contribute to the task-related ERPs as measured on the scalp. The results indicated that the right SMG contributes to the location-related effect on the N1 component at right posterior sites. Most interestingly, the right SMG activated in the fMRI is consistent with the N1 source analysis. Since four reference data types showed the location effect at the right posterior sites (Figure 4), the fMRI result here is insufficient to verify which reference is preferable.

Although, the ERP and source analysis showed a location effect on both sides of posterior areas, fMRI showed a location effect only on the right SMG. These incongruent results on ERP and BOLD measures are common in simultaneous EEG and fMRI studies (Bledowski et al., 2006). This is because the ERP and fMRI BOLD signal are related directly and indirectly to neural activity separately (Ogawa et al., 1990; Nunez and Silberstein, 2000; Logothetis et al., 2001). Thus, the different sensitivity of the two modalities leads to activity visible in one modality but invisible in the other modality (Nunez and Silberstein, 2000).

### In comparison with EEG-only study

In order to confirm the results in the present simultaneous EEG-fMRI study, we used the ERP data collected in the same task but without active fMRI recordings to evaluate the effects of different reference procedures on task-related ERP results.

The results showed that the location repeated enhancement was reflected at bilateral posterior N1 and the VWM load suppression was reflected at central P300, which is consistent with the ERP results in the present EEG-fMRI study when using AR and two REST references. SPSM and source analysis of the N1 component confirmed that the bilateral posterior sources contribute to the bilateral posterior scalp N1 component, showing location repeated enhancement effects. These results are more closer to the LM and two REST references data in the present EEG-fMRI study. In general, the two REST references provide more consistent results between the simultaneous EEG-fMRI study and the EEG-only study.

### The effectiveness of ERP result

In the present study, we found that the N1 amplitudes were insensitive to VWM load and the visual side of the presented stimulus, indicating that the N1 component may reflect a discriminative process rather than a sensory-perceptual process of attention allocation (Vogel and Luck, 2000). Furthermore, we observed larger N1 amplitudes for the object location repeated condition compared to the object location changed condition. This is in line with previous studies reporting repetition priming effects in posterior N1, such as enhanced N1 amplitudes for the repeated stimuli (Ji et al., 1998; Soldan et al., 2006; Frings and Groh-Bordin, 2007). This result can be interpreted as the attention-based rehearsal for the memorized location facilitated the perceptual process of the probe appearing at the same location (Awh et al., 2000; Jha, 2002).

The P300 component has been shown to be associated with memory retrieval, stimulus evaluation, decision making (e.g., whether template matching the test array matches the memorial representations or not; Murphy et al., 2009). We found that P300 amplitudes decreased with increasing VWM load, implying that more processing resources, which are related to memory retrieval and the comparison between the test array and the memorial representations, were required and thus less “central resources” remained (Kok, 2001; Pinal et al., 2014). These findings are consistent with a previous study showing larger P300 amplitudes in VWM load 1 compared to that in load 3 (Bledowski et al., 2006).

## Conclusion

We found that the adopted reference did not change the topography map of N1 and P300 components, but it did alter the task-related effect on ERP components. LM decreased or eliminated the visual working memory (VWM) load effect on P300, and the AR distorted the distribution of VWM location-related effect at left posterior electrodes as shown in the SPSM of N1. For the RESTs and RESTr, they both revealed object location effects on N1 and VWM load effects on P300. The SPSM of N1 revealed a bilateral posterior distribution. This result is consistent with the source analysis (e.g., source distribution and source activities) of N1, which estimates are independent of the adopted references (e.g., AR, LM, RESTs, and RESTr). Furthermore, in comparison with the EEG-only study, the two REST references in the EEG-fMRI study provide closer results to the independent ERP study. Taken together, the two REST references revealed more integrated and reasonable results than AR and LM in the EEG-fMRI study. Furthermore, with the data of individual structured MRI and realistic electrode positions, we recommend the RESTr as the reference method for EEG data in the simultaneous EEG-fMRI study.

## Limitations

Previous studies have reported the effects of reference choice on EEG band analysis, using measures of power spectra, coherence, and network connectivity (Yao et al., 2005; Marzetti et al., 2007; Qin et al., 2010; Chella et al., 2016). However, in our practical experiment, we are only interested in the task-related effect on ERP and fMRI results, which is a major concern in cognitive neuroscience research. Thus, we care more if the reference method would alter the “what” and “where” of task-related effects on ERP components.

We hoped to use the task-related fMRI activations to inform the ERP sources, and in turn to verify the distribution of the task-related effect measured on the scalp (ERP). Due to the sensitivity difference of the two modalities in neuronal activity, the experimental effect was detected at bilateral posterior electrodes for ERP but only at right posterior cortex for fMRI. Since four reference data types showed the location effect at the right posterior (Figure 4), the fMRI result here is insufficient to verify which reference is the best option.

The independent ERP study used a 128-channel EGI system, which is inconsistent with the 64-channel Neuroscan system used in the EEG-fMRI study. This will not provide the precise pairwise comparison between these two studies. However, the independent ERP study provides the what and where of task-related ERP effects that can help to evaluate the closest results in the EEG-fMRI study when using different reference methods.

## Author contributions

PY and LL conceived and designed the experiments. PY, CF, and MW performed the experiments and analyzed the data. PY wrote the main manuscript text. All authors reviewed the manuscript.

### Conflict of interest statement

The authors declare that the research was conducted in the absence of any commercial or financial relationships that could be construed as a potential conflict of interest. The handling Editor declared a shared affiliation, though no other collaboration, with the authors and states that the process nevertheless met the standards of a fair and objective review.
